# Aptamer-engineered (nano)materials for theranostic applications

**DOI:** 10.7150/thno.85419

**Published:** 2023-09-25

**Authors:** Navid Rabiee, Suxiang Chen, Sepideh Ahmadi, Rakesh N. Veedu

**Affiliations:** 1 Centre for Molecular Medicine and Innovative Therapeutics, Health Futures Institute, Murdoch University, Perth, WA 6150, Australia.; 2 Perron Institute for Neurological and Translational Science, Perth, WA 6009, Australia.; 3 Student Research Committee, Department of Medical Biotechnology, School of Advanced Technologies in Medicine, Shahid Beheshti University of Medical Sciences, Tehran, Iran.; 4 Cellular and Molecular Biology Research Center, Shahid Beheshti University of Medical Sciences, Tehran, Iran.

**Keywords:** nanomaterials, aptamer, aptamer modified materials, biosensors, biomedical engineering

## Abstract

A diverse array of organic and inorganic materials, including nanomaterials, has been extensively employed in multifunctional biomedical applications. These applications encompass drug/gene delivery, tissue engineering, biosensors, photodynamic and photothermal therapy, and combinatorial sciences. Surface and bulk engineering of these materials, by incorporating biomolecules and aptamers, offers several advantages such as decreased cytotoxicity, improved stability, enhanced selectivity/sensitivity toward specific targets, and expanded multifunctional capabilities. In this comprehensive review, we specifically focus on aptamer-modified engineered materials for diverse biomedical applications. We delve into their mechanisms, advantages, and challenges, and provide an in-depth analysis of relevant literature references. This critical evaluation aims to enhance the scientific community's understanding of this field and inspire new ideas for future research endeavors.

## Introduction

Nanomaterials refer to materials composed of small particles with sizes ranging from 1 to 100 nanometers. These materials possess unique properties that make them highly valuable in various applications, including medicine 1-3. For instance, lanthanide-doped upconversion nanoparticles (UCNPs) exhibit nonlinear light transformation, exceptional stability, and resistance to photobleaching. Magnetic nanomaterials demonstrate superparamagnetic characteristics, while carbon nanomaterials exhibit a broad range of absorbance 4-6. Metal-organic frameworks (MOFs) exhibit desirable features such as tunable porosity, appropriate size, and engineered morphology, making them well-suited for biomedical applications 7, 8. Liposomes, consisting of a lipid layer and a core for encapsulating drugs, are among the first nano-scale materials for drug delivery. By modifying the lipid layer, liposomes can fulfill multiple functions, enabling effective drug delivery 9. Polymer-based nanoparticles offer specific structures tailored for various applications, including gene/drug delivery 10, 11. One commonly used polymer nanoparticle is polylactic-co-glycolic acid (PLGA), which demonstrates excellent compatibility and degradation properties, making it widely utilized as a drug carrier for cancer therapy. However, PLGA does have limitations in terms of stability. Dendrimers, on the other hand, are versatile and biocompatible macromolecules with functional groups on their surface that enhance their ability to deliver therapeutic drugs 12, 13.

Recently, there has been a shift in (nano)material applications from a laboratory setting to a cellular level, with applications including imaging, drug/gene delivery, and cancer therapy. However, many nanomaterials tend to accumulate non-specifically in tissues due to the enhanced permeability and retention (EPR) effect and lack the capability to selectively target specific regions of interest (ROI). As a result, the potential toxicity of nanoparticles in non-target tissues has limited their applications in biological fields 14, 15. To overcome these limitations, various techniques have been used to decorate nanomaterials with molecules to enhance their selectivity.

**Scheme 1:**
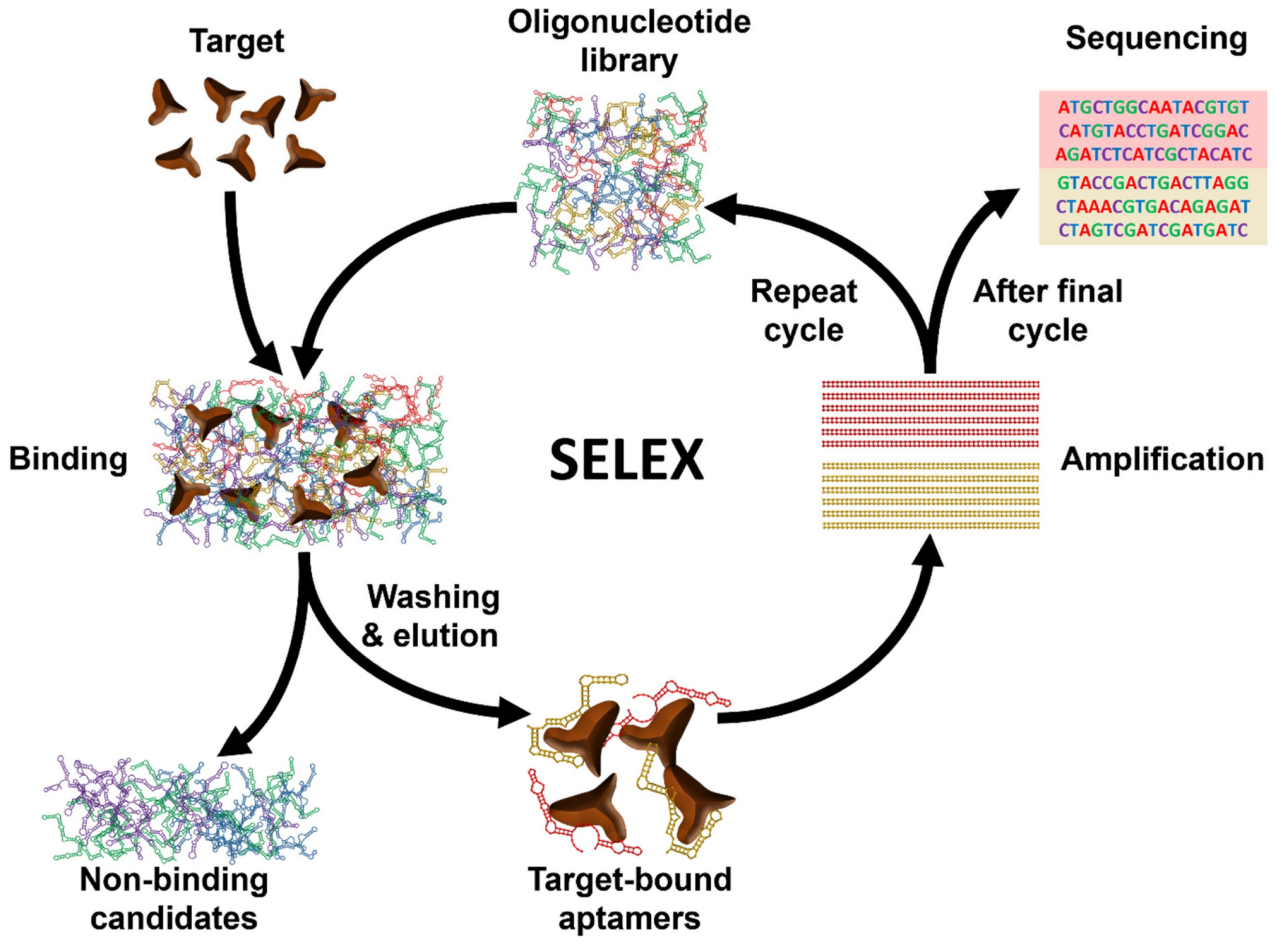
Schematic illustration of the general process of Systematic Evolution of Ligands by EXponential enrichment (SELEX).

Aptamers are short single-stranded (ss) RNA or DNA molecules identified through the Systematic Evolution of Ligands by EXponential enrichment (SELEX) process (Scheme 1). The SELEX method involves the selection of nucleic acid sequences comprising variable regions of 30 to 60 nucleotides, flanked by fixed regions of 15 to 30 nucleotides on each end, to form the starting pool. These oligonucleotides are incubated with the target molecule, and the sequences that bind to the target are isolated, purified, and amplified through polymerase chain reaction (PCR). This iterative process is repeated multiple times to refine and enrich the sequences that demonstrate affinity for the target. For DNA aptamers, the initial incubation requires ssDNA. To achieve this, the 3' primer is biotinylated, and it is separated from the complementary strand using streptavidin beads. The purified DNA sequences from each round are utilized for subsequent iterations. To isolate RNA aptamers, a 5' primer containing a T7 RNA polymerase promoter is first annealed with a 3' primer. Double-stranded (ds) DNA is then generated through extension using the Klenow fragment or multiple rounds of PCR. This dsDNA is transcribed into ssRNA, which interacts with the target. Subsequently, the 3' primer is annealed to the RNA in the presence of a transcriptase enzyme to synthesize complementary DNA (cDNA). This cDNA is then amplified through PCR to generate dsDNA 16.

Aptamers can bind to an extensive range of targets, including proteins, ions, and biomolecules with great affinity 17, 18. Aptamers offer numerous advantages over antibodies. These benefits include high reproducibility, low molecular weight, small size, and absence of immunogenicity or toxicity 19, 20. Aptamers, in particular, have become popular due to their capacity of highly selective target recognition 21-24. The nature of nucleic acids also makes aptamers useful in biological applications. Aptamer sequence can be modified to allow tunable recognition of targets. Aptamers have been explored for use as sensing tools for drug monitoring and disease diagnosis, and also for use in therapeutics either through targeted delivery of therapeutic cargo into cells or regulating protein function to influence biological processes 25-27. Although several aptamer-based drugs are now in clinical testing, one, Pegaptanib, is already on the market to treat age-related macular degeneration (AMD). However, natural nucleic acid aptamers can be degraded by nucleases in complex environments, which restricts their use. To address this issue, aptamers can be conjugated to carriers to increase their resistance to nucleases. Aptamers offer the advantage of being easily modified with functional groups, allowing for their attachment to nanomaterials and manipulation upon target binding 28, 29. By introducing an antidote, the conformation of an aptamer can be altered, resulting in release of the target. This technology has been successfully utilized to control anticoagulation levels in patients requiring anticoagulation therapy. Aptamers can be chemically synthesized, enabling convenient functional group attachment at their ends 30. It is worth noting that many benefits that are attributed to aptamers, such as their appropriate size and capacity for functionalization with various groups, are shared by other targeting molecules, including small antibodies and biomolecules 31. Consequently, careful consideration should be given to selecting the most suitable targeting biomolecule for a specific application, considering scientific and practical factors. Scientific considerations include factors like charge, size, binding affinity, and ligand stability. In the case of antibodies, it is also crucial to consider their potential to trigger antibody-dependent cell-mediated cytotoxicity 32, 33. Aptamers have further been incorporated into the development of nanomaterials for drug delivery purposes within biological environments 34-37.

One of the main challenges in using aptamers in bioapplications is their relatively short serum half-lives, which can limit their effectiveness. Researchers have developed several strategies to address this issue, including using aptamer conjugates and developing new SELEX techniques. Aptamer conjugates are aptamers that are chemically linked to a carrier molecule, which can increase their stability and thereby extend their serum half-lives. In fact, the molecular weight of aptamers (<20 kDa) is lower than the renal filtration threshold (50 kDa), causing a short half-life, which restricts their potential use as therapeutic agents. (Nano)materials and biomolecules can be developed for enhanced drug loading, increased half-life in the body, and selective distribution by modifying their composition, size, and morphology 38. PEGylation can reduce the bottleneck in developing aptamers into therapeutics, through extending their circulation half-life and reducing nanoparticle aggregation 39, 40. One study showed that Apt-paclitaxel (PTX)-NP exhibited a much longer elimination half-life and slower clearance rate than PTX-Apt 41. PLGA/PEG nanoparticles can protect aptamer from degradation and increase circulation time of Apt-nanoparticles (half-life of Apt-PTX-NP was 4h). Aptamer-conjugated chitosan nanoparticles can considerably increase the efficiency of traditional therapies, decrease their side effects on normal tissues, and overwhelm the enhanced EPR effect caused by the nanomaterial. Besides, these conjugations help to increase their half-life in bodily fluids via enhancing the nuclease resistance of aptamers 42. Another study showed the stability and high half-life of A3-APO^His^ during 28 h, which is higher than other associated peptides. A3-APO^His^ was not observed in the organs of mice injected with APO^His^ alone, AuNP-Apt^His^ significantly increased the cell penetrating capability of A3-APO^His^ in mice 43.

Novel SELEX techniques, such as modified SELEX (mod-SELEX) and circular SELEX (cSELEX), have also been established to enhance the stability of aptamers 44. One of the most promising areas of research in aptamer nanotechnology is drug delivery. Aptamers can be designed to target cancer cells, diseased tissue, or proteins, making them ideal candidates for delivering therapeutic cargo to specific locations in the body. Apart from its role in drug delivery, aptamers can also be used to directly regulate protein function, which can influence vigorous biological procedures such as immune stimulation pathways. As mentioned earlier, some therapeutic aptamers are currently under clinical investigation, and Pegaptanib was approved by the US Food and Drug Administration (FDA) to treat AMD. Furthermore, aptamers are used for imaging and diagnosis. Aptamers can be labeled with various imaging agents, such as fluorescence dyes or radioisotopes, which can be used to visualize specific targets in the body, thereby benefiting disease progression monitoring and cancer therapy 45-47.

The Cell-SELEX process aims to recognize aptamers that specifically bind to a particular cell type. The Cell-SELEX method can be applied to generate aptamers that are capable of targeting nanoparticles to tissues of interest by identifying unique molecular features of targeted cells. For example, Cell-SELEX has been applied to isolate an aptamer that targets glioblastoma, a type of brain cancer 48. Cell-SELEX has also been used to isolate aptamers that prevent the reorganized throughout transfection (RET) receptor tyrosine kinase, which is involved in cancers. Aptamers selected using the Cell-SELEX method may be capable of enhancing the targeting of nanoparticle-aptamer conjugates to targeted cells, as well as potentially improving the intracellular delivery of nanoparticles. In fact, the selection process can enrich the set of aptamers that can escape endosomal degradation. While aptamers that facilitate cytosolic delivery have the potential to be used in conjunction with nanomaterials, their function in this context would still need to be validated and confirmed. Researchers are also exploring approaches to deliver aptamers across the cell membrane, which could lead to innovative applications of nanotechnology. The Tan group used Cell-SELEX to develop aptamer molecular probes for identifying, analyzing, and isolating tumor cells. They recognized a group of aptamers connecting to a T-cell acute lymphoblastic leukemia cell line. These aptamers, labeled with fluorescence dyes and analyzed using flow cytometry, allowed the team to identify target cells inside samples like bone-marrow aspirates. They also confirmed that the aptamers were internalized by leukemic T cells 49-51.

Although a number of review articles have been published about aptamer-conjugated materials in theranostic applications 52-54, the present work provides a broad perspective on aptamer-conjugated (nano)materials, including organic and inorganic materials, and their different theranostic applications, such as diagnosis, gene/drug delivery, and cancer therapy. Finally, we provide an in-depth discussion about the challenge of these aptamer-nanomaterial systems along with casting a significant eye over the issue.

## Aptamer-assisted nanotechnology

Aptamers have many potential applications in nanotechnology to treat diseases. They are particularly useful in this context due to their small size, allowing them to be used in drug delivery devices without significantly increasing the device's overall size and allowing for cell or tissue selectivity 55. Aptamers potentially work for a large range of potential drug delivery due to their capability to connect to various targets, such as hepatitis C virus proteins, thrombin, HIV-1, and human thyroid-stimulating hormone. Aptamers can be applied to block antibodies that bind to the insulin receptor, which could interfere with the treatment of insulin resistance. Aptamers capable of binding to vascular endothelial growth factor (VEGF) can play a significant role in anti-angiogenesis. This aptamer has received FDA approval to treat AMD. Aptamers can act as inhibitors themselves without requiring related toxins, but long-term data is required to measure their toxicity when injected intravenously 56, 57.

Drug-loaded nanoparticles have shown promising results in preclinical studies. Conjugating aptamers to nanoparticles can lead to targeting specific cells with increased precision, thus improving efficacy of therapeutics and specificity of diagnosis. For example, the A10 aptamer has been conjugated into nanoparticles and used to target the prostate-specific membrane antigen (PSMA), a transmembrane protein overexpressed in prostate cancer. This platform was investigated in the lateral tumor model of LNCaP prostate cancer, and the effective reduction of tumor size was observed after intratumoral injection 58. Aptamer-toxin conjugates can be used as a therapeutic agent. For example, conjugating the A9 aptamer with Gelonin as a ribosomal toxin caused 600-fold improved potential in cell death in cells expressing PSMA and reduced toxicity in non-targeted cells 59. Aptamers have also been applied to release anthracycline chemotherapeutics and to create quantum dot-aptamer conjugates that can identify cancer cells and determine if a drug has been delivered. By fixing aptamers on carbon nanotubes, the presence of analytes can be detected, and they can be used to create smart nanostructures to detect analytes 60-62.

### Conjugation strategies

Nanomaterials could be modified with various kinds of aptamers as ligands. These modifications are attained by different methods, such as covalent bonds and physical conjugation approaches which were applied via a linker to preserve aptamer binding activity. In nanomaterials, their surface allows the anchoring of different aptamers. Besides, aptamers have terminal functional groups with high flexibility. Covalent and non-covalent strategies are the two most common techniques.

#### Non-covalent strategies

In this type of strategy, electrostatic interactions, hydrogen bonding interactions, π-π interactions, and van der Waals interactions are involved in the formation of bonds between aptamer and nanomaterials 63. DNA bases can use π-π stacking and hydrogen bonds to interact with graphene oxide (GO) 64. Based on these strategies, some effective modifications are established, such as using imidazolium ring groups to offer self-assembly properties and biotin-avidin interactions 65, 66. Direct self-assembly of DNA into nanostructures has different applications. For example, conjugation of aptamers on avidin-liposomes was performed through avidin-biotin interaction, where an anti-platelet-derived growth factor receptor aptamer was connected to DOX-liposomes sensitized using poly (NIPMAM-co-NIPAM) 67.

#### Covalent strategies

Covalent strategies are more often applied in the modification of nanomaterials, since they are more stable than the non-covalent strategies. Therefore, covalent interactions are broadly applied to the synthesis of nanomaterials with various functional groups 68. Aptamers can be attached directly to the surface of nanoparticles, although some scientists use linkers to connect aptamers by covalent bonds. Thiol is the linker for gold (Au) nanoparticles to connect a biomolecule with the nanoparticles. Thiol maleimide coupling chemistry is generally used for the conjugation of thiolated (-SH) molecules to the surface of nanomaterials in drug delivery systems 69. Hydroxyl, Amine, and carboxylic acid are other groups on the surface of nanomaterials. The aptamer conjugation was attained through carbodiimide chemistry, where the D-ɑ-tocopheryl polyethylene glycol succinate (TPGS) polymer was treated with succinic anhydride to attain carboxyl-modified polymer, and an amine-modified AS1411 aptamer was added to the EDC/NHS-activated TPGS polymer. This platform showed greater cellular uptake and major cytotoxicity 70. However, these moieties are solvent-exposed to react, and these approaches for modification are not suitable. Some illustrative studies are demonstrated as follows. Amine moieties can be reacted with p-isothiocyanate or N-hydroxy-succinimide esters for functionalization 71. Nanomaterials with hydroxyl groups can be modified with carbonyldiimidazole to form a reactive intermediate. Besides the epoxy groups can be used in function with aptamers, which are applied for amine groups-contained aptamers 72.

## Aptamer-embedded DNA (nano)materials

In addition to their role in transmitting genetic information, DNA can be used as molecular building blocks to form various types of (nano)materials with manageable sizes, shapes, and functions based on Watson-Crick hybridization, enabling the progress of DNA nanotechnology. Aptamers can be integrated into DNA (nano)materials, creating aptamer-embedded DNA (nano)materials including DNA nanostructures, DNA-based micelles/polymers, DNA hydrogels, and DNA-functionalized liposomes. These (nano)materials have shown great potential for biomedical applications 73.

### DNA nanostructures

DNA nanostructures are nanoscale structures formed of DNA, which acts as a functional element. In fact, they can act as scaffolds for the fabrication of complex structures 74. DNA nanostructures have been widely applied for the management of biological procedures, which is necessary in investigating the molecular mechanism of biomedicine. DNA nanostructures have several applications in biosensing, treatment, and therapeutic agent delivery. As a result, modified DNA structures with theranostic moieties were applied for the targeting of different immunological, cancer, and metabolic diseases 75, 76.

**Figure 1:**
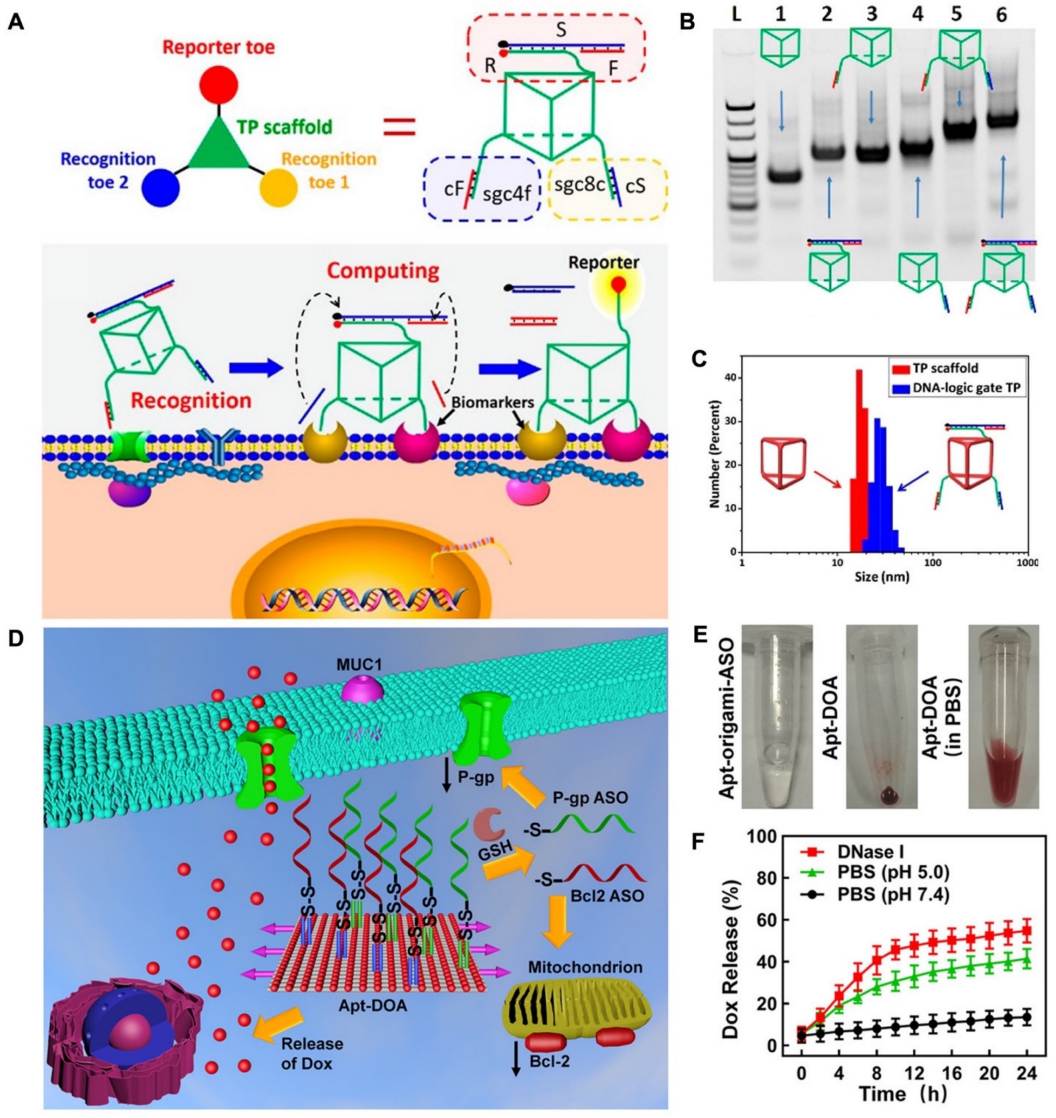
Working principles of engineered DNA nanomachine. (A) Schematic illustration of the working mechanism of DNA-based nanomachine. Structure of DNA-based nanomachine, and the aptamer DNA nanomachine for cell surface computing: the binding of two aptamers to their biomarkers and releasing cS and cF from recognition toes. (B) PAGE results established the assembly of the DNA-logic gate TP. Lane 1: DNA TP scaffold. Lane 2: F/S/R-TP. Lane 3: sgc8c/cS-TP. Lane 4: sgc4f/cF-TP. Lane 5: sgc8c/cS-sgc4f/cF-TP. Lane 6: F/S/R-sgc8c/ cS-sgc4f/cF-TP. (C) Dynamic light scattering (DLS) results for determination of the size of 500 nM TP scaffold (red) and DNA-logic gate TP (blue). Reprinted (adapted) with permission from 78. Copyright 2018 American Chemical Society. (D) The design of Apt-DOA with 12 MUC1 aptamers, Bcl2, and 28 P-gp ASOs, targeted co-delivery of ASO and DOX to improve therapy in drug-resistant cancer cells. (E) Schematic illustration of the synthesized Apt-origami-ASO dispersed in PBS buffer. (F) Dox-release profile of Apt-DOA in PBS buffer. Reprinted (adapted) with permission from 91. Copyright 2020 American Chemical Society.

The unique capability of aptamers to identify and connect to cancer cells makes them valuable apparatuses for precise cancer treatment. This is because aptamers can be combined into DNA nanostructures through hydrogen bonding interactions. This allows for specific cell recognition and following applications. For instance, researchers developed an aptamer-modified DNA structure, "Nano-Claw", that is capable of investigating cancerous cells and being utilized in targeted therapy 77. Peng and colleagues developed a 3D DNA nanomachine that was designed for specific targeting of biomarkers. The nanomachine was composed of a DNA of triangular shape with prolonged toes on the top and both sides of faces (Figure 1A). The toes on the top face performed as reporters and were made up of three strands: S, F, and R. On the bottom face, two separate recognition toes performed as an "AND" Boolean operator. As each edge of the triangle showed a single-strand domain for hybridizing functional toes (Figure 1B), a toes-loaded DNA nanomachine was developed. The nanomachine was able to recognize and bind to two types of membrane biomarkers that were overexpressed on CCRF-CEM cells, and the AND operator returned an accurate value, activating the reporter toes via DNA displacement. The nanomachine demonstrated improved molecular recognition compared to other molecular paths based on dsDNA and could accurately recognize specific cell subtypes 78.

Researchers developed a DNA logic device that activated aptamers via hybridization reaction to precisely recognize specific cells from a large population of similar cells 79. Multiple aptamers that target membrane receptors can be applied. A toehold-based reaction occurs by simultaneously binding several aptamers to cells, which enables the detection and amplification of the signal from the target cells. Also, these aptamer-decorated DNA nanostructures can be used as smart biosensors to measure intracellular biomolecules for cellular interactions 80, 81. Li and colleagues presented the development of a DNA probe that is compatible, efficient, and adaptable in its ability to use cell connections. The probe's design incorporated the structural rigidity of 3D pyramidal DNA. Compared to linear DNA probes, the pyramidal probes demonstrated significantly enhanced stability for cell membrane anchoring, with an approximately 100-fold increase. Additionally, the pyramidal probes showed a 2.5-fold increase in target accessibility between two different kinds of cells. Using these probes allowed the researchers to investigate the role that close proximity plays in cell interactions, and they found that it is crucial. Therefore, this approach enables nanoplatforms to study cell membrane anchoring in cell communication networks 81.

Scientists have used molecular engineering techniques to create DNA nanostructures with aptamers embedded in them to carry therapeutic agents to specific cells or areas of the body. One example of this is the use of aptamer-based nano assemblies that can target certain types of exosomes (tiny vesicles released by cells) while ignoring others, allowing for the accurate delivery of DNA nano assemblies to specific organelles (tiny structures within cells) 82, 83. In 2013, a group of researchers led by Zhu created aptamer-tethered DNA nanotrains (aptNTrs) to deliver doxorubicin (DOX) to specific cells. These aptNTrs used aptamers to target the cells 84. aptNTrs were self-assembled from two DNA commenced by aptamers, which act as locomotives train toward tumors. sgc8 was internalized by target CEM cells through endocytosis. aptNTrs worked as carriers with a great payload capacity of drugs that were transported to target cells and prompted cytotoxicity. In fact, aptNTrs increased the maximum -tolerated dose in nontarget cells. More recently, a group of researchers led by Xue created a DNA nanowire (NW) to target cancer cells for imaging and therapy. This NW consisted of multiple binding DNA helices assembled using two structural units, with terminal-hidden aptamers on the surface that recognize tumor cells. Aptamer-DOX DNA NWs can enter the blood circulation through endocytosis of cells in blood and are able to accumulate in target tissues. The aptamer can bind to the membrane protein tyrosine kinase-7 (PTK7) overexpressed by cells and it can fold into a hairpin structure 85. Another group of researchers, led by Ouyang, developed a DNA nanoscale precision-guided missile (D-PGM) for the targeted delivery of therapeutic agents to target cells, to improve the effectiveness of treatment. The D-PGM is formed with a DNA structure for loading the therapeutic agents and a control system to achieve a great payload. They used aptamers bound to target cells as an "initiator" for the guidance/control (GC) system of the D-PGM. The GC system is based on an aptamer-based logic gate, and the warhead (WH) is a DNA self-assembled 3D structure containing a therapeutic agent called DOX. When the D-PGM reaches the tumor tissue, the GC system can be disassembled, allowing the D-PGM to bind to and be taken up by target CEM cells, providing a more efficient drug delivery system and lowering toxicity to non-target cells. Li and co-workers created a drug delivery system called Apt-ND-ABP, consisting of a backbone made of miR-21 and miR-150 and easily loaded with small RNA molecules such as siRNA or miRNA 86, 87. When the Apt-ND-ABP binds to A549 cells, it is taken up into the cell's cytoplasm. It activates the release of multiple antisense oligonucleotide, preventing the function of specific miRNAs and the controlled death of the target cells (apoptosis). Another group of researchers, led by Ren, used a lock-and-key technique to precisely deliver siRNA to specific cells. They employed two DNA aptamers, sgc8c, and sgc4f, that bind to the cell membrane of target CEM cells as "double locks" and an oligonucleotide nanovehicle (ONV) functionalized with a hairpin construction as the "smart key". The "lock" can be opened after hybridization and cleavage of the hairpin, enabling the release of siRNA into the cells 88.

DNA origami, a DNA-based nanostructure composed of multiple building blocks, has played a significant role in advancing the field of DNA nanotechnology, particularly in biomedical applications. This is due to the ability of DNA origami to be functionalized with various groups, including functional nucleic acids. By incorporating nucleic acid aptamers into DNA origami, targeted delivery of cargo can be achieved, enhancing its potential in biomedical applications 89. In recent developments, researchers have successfully created a DNA nanorobot designed for intelligent drug delivery. This nanorobot is engineered to carry the enzyme thrombin within its structure. On the surface of the nanorobot, an aptamer known as AS1411 is introduced, which specifically binds to a protein called nucleolin that is expressed in tumor cells. Upon reaching the targeted tumor site, the DNA nanorobot can be triggered to open and release the thrombin payload. The released thrombin then initiates blood clotting processes, resulting in tumor necrosis and suppression of tumor growth. This innovative approach holds great promise for safe and precise drug delivery in cancer therapy, offering potential advancements in the field 90. While several studies have shown the efficacy of using DNA origami nanostructures for drug delivery, there is still potential for further exploration of their use in therapeutics as the building blocks of origami. In a study, researchers used nanocarriers with DNA origami (Apt-DOX-origami-ASO) to deliver chemotherapy drugs and antisense oligonucleotides (ASOs) for the treatment of drug-resistant cancer cells. The Apt-DOX-origami-ASO nanocarrier developed by Pan et al. consists of a DNA origami structure that is functionalized with staple strands protracted with MUC1 aptamer, which gives it targeting capability. The origami can carry ASOs by strand hybridization and has the DOX loaded onto it through electrostatic adsorption. This nanocarrier showed promising results in terms of controlled drug release and gene silencing (Figure 1D-F) 91.

DNA nanostructures are typically made up of many nucleic acid (NA) sequences, which result in a great number of intrinsic nicks in the phosphodiester bonds of the DNA. These nicks increase the instability of the DNA structures by providing more high sites for cleavage by nucleases. To address this issue, researchers have developed strategies for constructing DNA nanostructures using a few DNA strands, reducing the number of potential cleavage sites and increasing the structures' stability. In 2013, a group of researchers led by Zhu established self-assembled nanoflowers (NFs) by the rolling circle amplification (RCA) technique. These NFs are made up of non-nicked building blocks that are densely functionalized, which allows them to be prepared using only a few DNA strands. This design overcomes the problems of intrinsic nicks that can affect the stability of the nanostructures and the complexity of their preparation. NFs are attractive biomaterials due to their ease of synthesis and good compatibility, and they have been explored for various biological applications 92, 93. Scientists have developed a DNA NF that can be used to deliver drugs specifically to cancer cells. These NFs, called Sgc8-NFs-Fc, are made of artificial analogues, and can be made in sizes ranging from 50 to 1000 nanometers. They can be degraded and release the drug they carry when exposed to hydrogen peroxide. They also comprise aptamers, which allow them to bind to and enter cancer cells. In both lab and animal studies, the Sgc8-NFs-Fc NFs showed good targeting efficiency against tumors, making them a promising tool for the cancer drug delivery 94.

### DNA-based micelles/polymer

Micelles are self-assembled molecules with a size ranging from 10 to 100 nm. They consist of a hydrophilic shell and a hydrophobic core, making them suitable for drug incorporation. The hydrophilic shell serves to prevent drug loss and evade the opsonization process triggered by the complement system, which otherwise leads to the rapid clearance of drugs from systemic circulation 95, 96. Scientists have successfully developed a nanostructure known as spherical DNA micelle, composed of amphiphilic oligonucleotides that can undergo self-assembly. These DNA micelles exhibit a multivalent effect, which significantly enhances their capacity to bind to specific targets using aptamers. As a result, they hold great potential for applications in drug/gene delivery systems and biosensors 97. Researchers have developed a technique for producing targeted aptamer-lipid micelles by connecting aptamer and lipid compounds using a methacrylamide branch. When exposed to adequate light, these components form a covalent bond, resulting in the formation of aptamer-lipid micelles. This innovative approach enhances the stability of the micelles and opens up possibilities for their utilization in imaging applications 98. In another study, researchers designed aptamer-based micelles for cancer-targeted chemodynamic therapy (CDT). These micelles are made of amphiphilic oligonucleotides and contain hydrophobic prodrug bases. When activated, they generate toxic radicals in cancer cells. This approach offers a new method for designing aptamer-based micelles for cancer therapy. It overcomes the high dependence on tumorous hydrogen peroxide and the strong acidity required for classical Fenton or Haber-Weiss chemistry in CDT 99.

**Figure 2:**
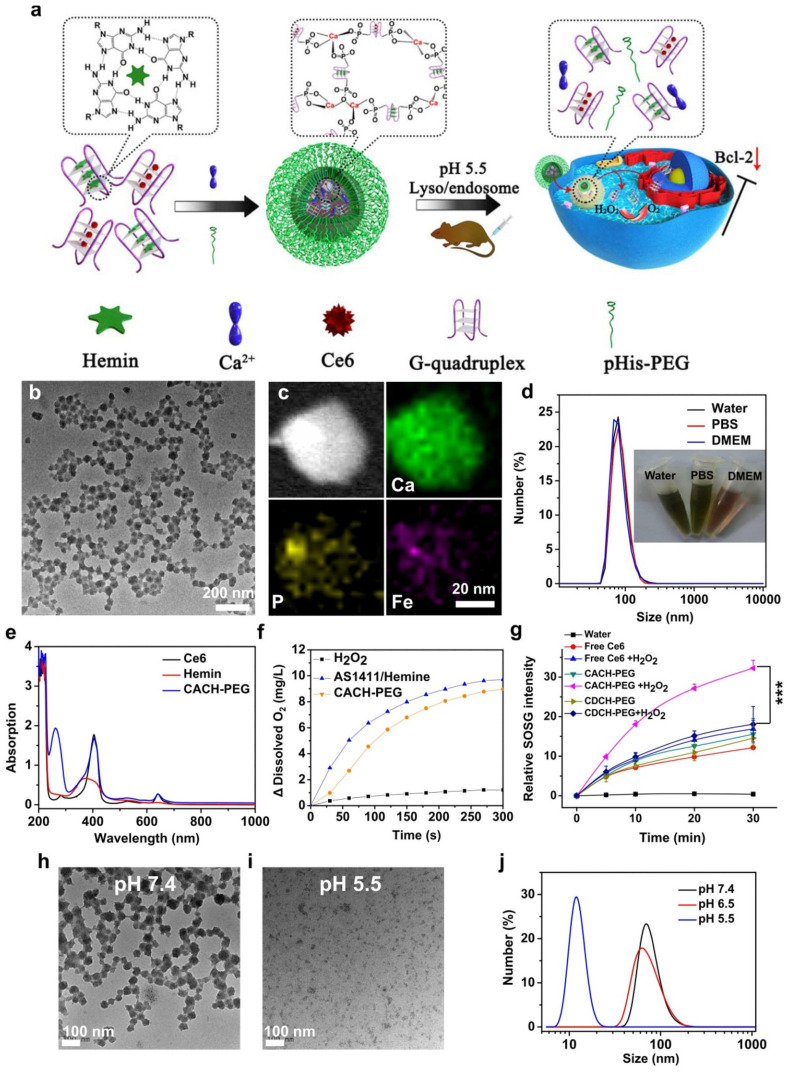
Synthesis and properties of Ca-AS1411/Ce6/hemin@pHis-PEG Nanocomplexes (CACH-PEG). (a) Illustration depicting the preparation of CACH-PEG. (b, c) Transmission Electron Microscopy (TEM) image (b) and Scanning Transmission Electron Microscopy (STEM) mapping (c) of CACH-PEG Nanocomplexes. (d) Hydrodynamic sizes measured by Dynamic Light Scattering (DLS) and a photograph (inset) of CACH-PEG dispersed in water, PBS, buffer, and DMEM cell-culture medium. (e) UV-visible-near-infrared (UV-vis-NIR) spectra of Ce6, hemin, and CACH-PEG. (f) Generation of oxygen in 2 mM H2O2 solutions after adding AS1411/hemin (AH) complex or CACH-PEG Nanocomplexes at room temperature. (g) Light-triggered generation of singlet oxygen measured by increased SOSG fluorescence for free Ce6 CDCH-PEG or CACH-PEG under 660 nm light irradiation in the absence or presence of H2O2. (h, i) TEM images of CACH-PEG after overnight incubation in PBS at (h) pH 7.4 or (i) pH 5.5. (j) Hydrodynamic sizes of CACH-PEG after incubation in PBS at pH 7.4, 6.5, or 5.5 for 1 hour. Reprinted (adapted) with permission from 102. Copyright 2018 American Chemical Society.

Among the various nanomaterials, polymers have garnered significant attention due to their structural diversity, allowing for the attainment of different sizes, morphologies, and desirable surface properties. Polymers can be employed as imaging agents, offering advantages such as long half-life, high stability, compatibility, and increased tissue density 100. Biodegradable polymer-based nanomaterials propose appropriate applications in the drug/gene delivery systems, cancer therapy, and biomedical fields 101. By replacing the hydrophobic portion of oligonucleotides with polymers, including poly(d,l-glycolic acid) and poly(d,l-lactic acid), researchers have created multifunctional polymer nanostructures that can be loaded with drugs. These nanostructures have potential applications in biotechnology. Researchers have developed nanostructured coordination polymers (NCPs) for use in photodynamic therapy (PDT). These polymers are composed of aptamer AS1411 and contain photosensitizer chlorine e6 and deoxyribozyme hemin. They have been modified with polyethylene glycol (PEG) and are referred to as Ca-AS1411/Ce6/hemin@pHis-PEG (CACH-PEG) NCP nanostructures. Studies have shown that coordination polymers with combined several therapeutic elements, such as CACH-PEG, can bind to and internalize into the nucleus of cells. To synthesize CACH-PEG, the AS1411 aptamer was first utilized to form a G quadruplex structure and loaded with Ce6 and hemin, resulting in AS1411/Ce6 with a size of less than 10 nm. Next, organic ligands AC and AH were mixed with CaCl_2_, along with pHisPEG as a stabilizing agent, to form CACH-PEG (Figure 2). The CACH-PEG nanostructure displayed a spherical morphology (Figure 2). Following the injection of CACH-PEG and subsequent imaging using a Lumina III *in vivo* imaging system, the accumulation of Ce_6_ signals in the tumor was observed after 8 hours, indicating the highly effective tumor retention of the CACH-PEG nanostructure. Furthermore, when mixed with ^99m^Tc, the CACH-PEG nanostructure could be chelated into the center of the porphyrin in Ce_6_, demonstrating excellent radiolabeling stability for *in vivo* applications 102. The G-quadruplexes and hemin within the polymer then exhibit DNAzyme activity, decomposing tumor endogenous hydrogen peroxide to generate oxygen to further reduce hypoxia-related resistance. These multiple therapeutic elements enhance the ability of the polymer to kill cancer cells.

Other researchers have designed DNA aptamer-hyperbranched polymers to accurately control drug delivery, which can enhance the biostability of aptamers. Aptamer-Polyprodrug conjugates have also been developed by connecting a compatible brushlike backbone to drug delivery systems. In a study, a ferrocene-comprising NA polymer for drug delivery against tumors was reported. The size of this polymer can be tuned through the Fenton-like reaction of ferrocene moieties in the tumor site, leading to size shrinkage down to 10 nm. The ferrocene loaded onto the NA polymer can release great toxic radicals to destroy tumor cells, improving the targeting efficiency of the polymer through increased permeability and enabling the nano-drug to penetrate deeper into tumors 102-105.

### DNA hydrogel

Functional DNA hydrogels, composed of DNA and possessing enhanced mechanical properties, have been developed for diverse biotechnology applications. These DNA hydrogels exhibit excellent water content and demonstrate high biocompatibility, rendering them suitable for a wide range of applications. Furthermore, the DNA modules integrated within the hydrogel exhibit unique recognition capabilities, enabling them to target biomarkers present on the surface of cells 106, 107. Recently, several studies have highlighted the significant potential of DNA hydrogels in cancer therapy. These hydrogels possess desirable characteristics such as biodegradability, compatibility, and programmability of DNA molecules 107. Besides, DNA hydrogel has high stability in the serum than other DNA structures. DNA hydrogel can be formed by simple RCA 108. Thus, they are considered to have potential in drug delivery systems according to their biocompatibility and stability. However, enhancement of large-scale chemical synthesis of DNA is required to reduce production costs. Generally, in order to avoid side effects, the biological properties of such DNA sequences should be considered in hydrogel design. This type of DNA hydrogels can be used as a very promising new biological material in medical applications.

These hydrogels can be engineered to respond to stimuli, enabling them to deliver functional cargo 109. For instance, researchers have developed stimuli-responsive and aptamer-based DNA hydrogels that can be applied for targeted gene regulation. Researchers have developed DNA nanohydrogels by using three compounds, including Y-shaped monomer B (YMB), Y-shaped monomer A (YMA), and a DNA linker. These compounds have three, one, and two sticky ends, respectively, which allow them to hybridize and form nanohydrogels. The size of these nanohydrogels can be controlled by adjusting the ratio of YMA to YMB. The researchers also incorporated aptamers and GSH-responsive linkages into the three units to fabricate aptamer-modified hydrogels that are applied for controlled gene delivery. These nanohydrogels showed effective internalization and high biocompatibility, exhibiting inhibition of cell proliferation with non-toxicity for normal cells. Besides, therapeutic genes can be effectively released from hydrogels for angiogenesis 110, 111. Researchers have developed hydrogels based on dual aptamers that can co-deliver two growth factors, VEGF and PDGF-BB, to promote angiogenesis. These hydrogels are assembled *in situ* after injection by aptamer-functionalized fibrinogen, which provides shelter for the delivery of the growth factors. Using multiple growth factors in this codelivery strategy is effective in promoting angiogenesis 112. In a separate study, a DNA poly-aptamer hydrogel was developed for gene therapy of cancer using CRISPR/Cas9 and immune checkpoint-blocking DNA aptamers. This hydrogel allows for the targeted delivery of gene editing and immune-modulatory agents to cancer cells, potentially providing a novel treatment method for cancer. A DNA aptamer hydrogel was created through an RCA process using a DNA strand with an aptamer against PD-1 and a sgRNA. The release of the PD-1 from the hydrogel was facilitated by the precise cutting action of Cas9/sgRNA, causing the obstruction of PD-1 and activation of the secretion of cytokine for splenocytes 113. This demonstrates the potential of hydrogel in immunotherapy.

**Figure 3:**
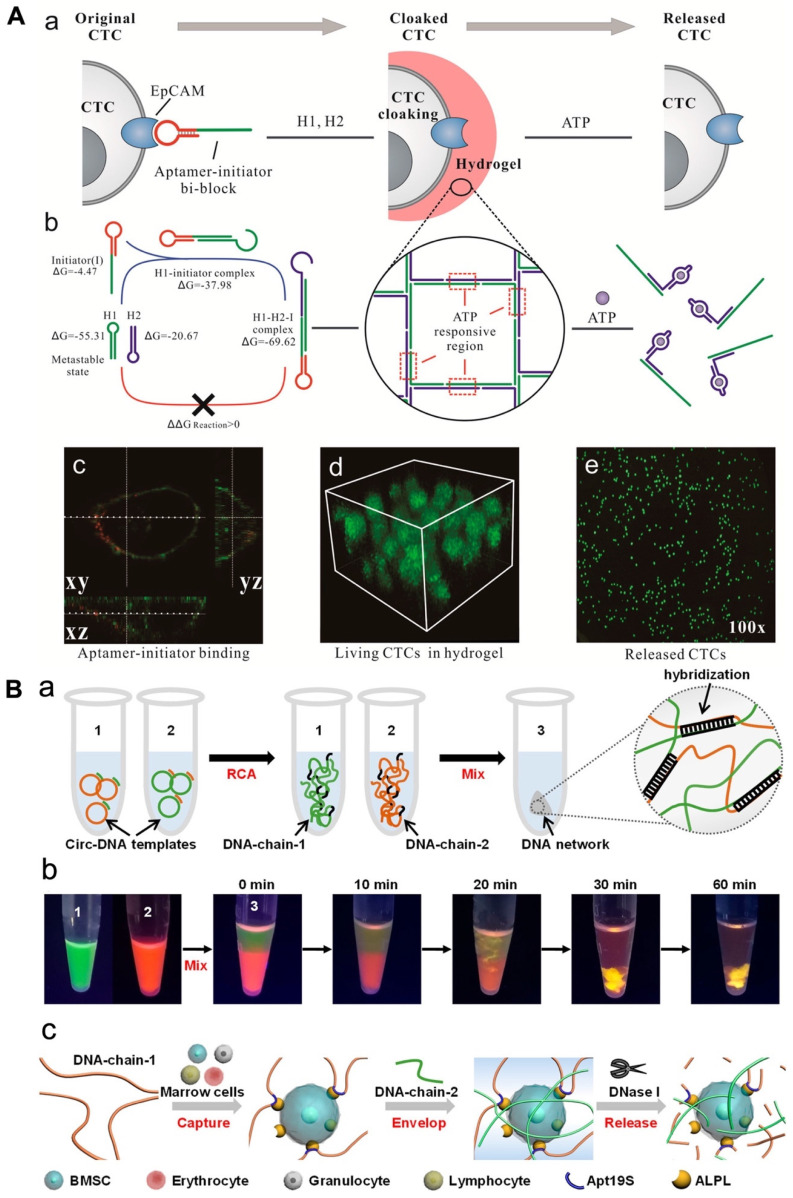
(A) DNA gelation-based cloaking and decloaking of CTCs. (a) The aptamer-initiator blocks were capable of binding to the EpCAM. (b) Confocal images of aptamer-initiator blocks (red) colocalized with DiO-stained lipid (green). (c) The 3D structure of MCF-7 cells is shrouded in DNA hydrogel, which displays multilayered cells in the hydrogel. (d and e) When ATP was added, the MCF-7 cells were released. Reprinted (adapted) with permission from 114. Copyright 2017 American Chemical Society. (B) (a) Design of a DNA network for stem cell fishing. (b) Formation procedure of DNA chains by RCA to attain a 3D network. (b) Combination of DNA chains to envision molecular diffusion throughout the fabrication of the DNA network. (c) The mechanism of capture includes capture, envelop and release. Reprinted (adapted) with permission from 115. Copyright 2020 American Chemical Society.

Researchers have explored the use of hydrogel based on aptamer to assemble cells, which could have applications in cancer diagnosis and cell-based therapies. In a recent study, a hydrogel based on aptamer was designed to capture circulating tumor cells (CTCs) using aptamer-triggered clamped hybridization chain reaction (atcHCR). This hydrogel can selectively capture CTCs from a blood sample, potentially enabling the detection and analysis of these cells for the diagnosis and treatment of cancer. A DNA strand made up of an EpCAM aptamer was used to recognize CTCs in a hydrogel using an atcHCR (Figure 3A). In fact, Figure 3 showed the binding of aptamer and EpCAM on the cell surface [cy3 (red) and DiO (green)]. The CTCs could be captured without significant damage and subsequently released for culturing and analysis by exposure to specific chemical stimuli 114. A separate study developed a DNA network to deliver bone marrow mesenchymal stem cells (BMSCs) without damaging the cells. This network was created through double RCA and self-assembly of two long DNA strands. The insertion of aptamers was done into DNA-strand-1 to allow to capture BMSCs, while DNA-strand-2 was applied to hybridize with chain-1 and fabricate a 3D structure to enclose the cells (Figure 3). The BMSCs could be released by digesting the DNA network with nuclease, with only minimal impact on cellular activity 114, 115.

### DNA-functionalized liposomes

In general, liposomes are small and spherical structures that have a cell membrane-like structure and a large cavity that can be loaded with various types of molecules and drugs 116. The multilayered spherical structures of liposomes with 50-500 nm in diameter particle size is greatly rich in lipid contents with different principles for their structural properties, their size and formation process, as well as drug loading 117. These properties make them appropriate for different applications, including drug/gene delivery, diagnosis, and cancer therapy 118-120. Theranostic dual-layered nanomaterial made by adding a liposomal layer to Au-PEG showed *in vivo* high stability. Functionalized nanomaterial is stable in physiological conditions, and the ^64^Cu labeled Limulus amebocyte lysate (LAL) platform displays sufficient blood circulation properties and an efficient tumor targeting capability of 16 %ID/g 118.

**Figure 4:**
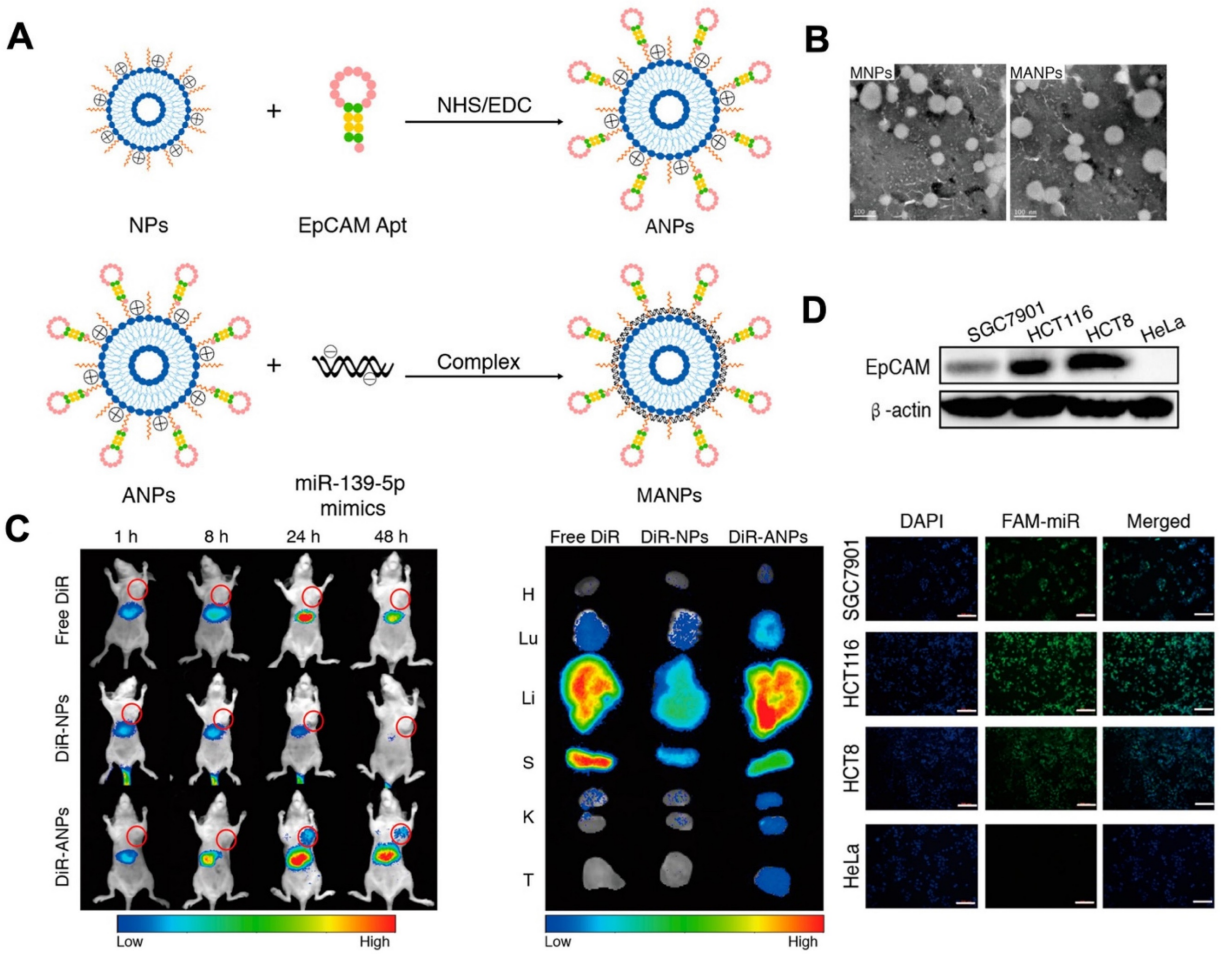
Schematic of the fabrication of NPs and ANPs. (A) Mechanism to form NPs and ANPs. (B) TEM image of MANPs and MNPs. The results showed the round-shaped morphology of liposomes. *In vivo* biodistribution of ANPs. (C) *In vivo* distribution of ANPs after intravenous injection for 1-48 h. (D) Fluorescence images of HCT116 cells, SGC7901 cells, and HeLa cells incubated with MANPs for 6 h. The scale bar is 200 μm. Reprinted (adapted) with permission from 121. Copyright 2019 American Chemical Society.

Nevertheless, the non-specific nature of cargo delivery by liposomes can be a limitation. For example, it can be difficult to deliver drugs via liposomes to targeted cells without any toxicity. In 2010, researchers established a liposome system based on aptamers for targeted drug delivery, which allows for the precise delivery of drugs to targeted cells while reducing the impact on normal cells. In this approach, sgc8 aptamers were used to enable the delivery of liposomes to target cells by specifically recognizing and binding to proteins on the surface of the target cell membrane. The use of aptamer modification allowed for greatly effective drug delivery. Recently, functional NAs have been packaged into liposomes for cancer therapy. Researchers established an aptamer-based liposome platform for the delivery of miRNAs. The platform was modified with EpCAM aptamers, demonstrating high effective internalization efficacy and inhibiting tumor growth (Figure 4A). The synthesis of liposome-aptamer was accomplished using the thin film hydration method. Subsequently, miRNA was loaded into the nanoparticles, specifically the EpCAM Apt-HSPC/DOTAP/Chol/DSPE-PEG2000-COOH (ANPs), through the self-assembly method. The results demonstrated that the liposomes synthesized exhibited a round appearance and dispersibility (Figure 4B). Furthermore, targeted delivery of the ANPs to colon cancer cells expressing EpCAM on HCT116 cells, Hela cells, and HCT8 cells was developed. It was observed that a stronger fluorescent signal was detected in HCT116 cells and HCT8 cells compared to HeLa cells, indicating the selective delivery of MANPs (Figure 4D) 121. As shown in Figure 4D, they observed the signal of DiR-ANPs in tumor tissues at 24 h. Furthermore, the signal of DiR-ANP stayed in the tumor for 2 days after the intravenous injection, while no signal was observed in the other groups. These nanoplatforms have the potential to perform as effective carriers to increase targeted therapeutic abilities. In addition to traditional chemical liposomes, mimetic liposomes made by extruding or secreting cells can also be prepared with aptamers for use in therapeutics. A delivery platform was developed with drugs, aptamers, and liposomes for cancer therapy. The biomimetic liposomes, derived from cells, were used to encapsulate and release drugs to cells through membrane fusion. This delivery platform effectively encapsulates and delivers a drug to the photodynamic and photothermal therapy 121-124.

Aptamer-based liposomes have the potential to release CRISPR/Cas9 complexes into specific cells for gene editing. For instance, researchers developed a liposome-CRISPR/Cas9 system based on aptamers for the delivery of sgRNA to permit the therapeutic application of the Cas9/sgRNA vector. This platform comprises an aptamer that binds to prostate cancer cells, which was able to decrease the expression of mRNA by approximately 60% *in vitro*, demonstrating significant cell-type binding ability. In addition, *in vivo* studies showed that gene silencing improved a noticeable deterioration of prostate cancer, providing future promise for the synthesis of aptamer liposome platforms for CRISPR/Cas9 delivery. Another study used an aptamer-modified lipopolymer to regulate the *VEGFA* gene in osteosarcoma. The aptamer LC09, which targets osteosarcoma, was applied to lipopolymer-containing CRISPR/Cas9 plasmids. Besides, LC09 can facilitate the sensitive distribution of CRISPR/Cas9 in orthotopic osteosarcoma and also inhibits osteosarcoma and lung metastasis. LC09-PPC-CRISPR/Cas9, also reduced expression of VEGFA and markers of proliferation and metastasis in lung metastatic sites 125-127.

## Aptamer-embedded inorganic nanomaterials

Nanomaterials that have been modified with specific aptamers have proven useful in different areas of biomedicine, such as biosensing, targeted drug delivery, cancer diagnosis, and treatment. This text will provide an overview of four inorganic nanomaterials with aptamers embedded in them, including carbon, gold, magnetic nanomaterials and metal-organic frameworks (MOFs). These materials have seen recent advancements in their use for diagnostic and therapeutic purposes.

### Gold-based nanomaterials

Gold nanomaterials have exceptional properties, and they can be used in bioapplications, including photoluminescence, light scattering, photothermal conversion, thermodynamic stability, biocompatibility, the ability to carry cargo, and the ability to be easily modified. These characteristics have led to using gold nanomaterials as building blocks in functional nanoplatforms 128, 129. For instance, aptamer-modified gold nanoparticles can be used as electrochemical and colorimetric biosensors for analysis. One study developed a sensor for the colorimetric detection of exosomal proteins using aptamers against exosome proteins against gold nanoparticle aggregation. In the existence of exosomes, the aptamers bind to proteins, leading to the release of free gold nanoparticles that rapidly aggregate and cause a color change that can be observed in a short time. This provides a quick sensing system for the early diagnosis of diseases 128. Another research has used aptamer-based gold nanomaterials to quantify intracellular adenosine triphosphate in cells and to efficiently kill cancerous cells through targeted drug delivery. Gold or its composites functionalized with aptamers have also been established for photothermal cancer therapy and cancer radiation therapy. These nanoplatforms can be applied as an effective approach for intracellular quantification of other molecules that use aptamer binding to remove a biological response 130.

PAMAM dendrimers, a type of synthetic polymer capable of encapsulating drugs and metal nanomaterials like gold nanoparticles, have been investigated by researchers for their theranostic potential. The study focused on a curcumin-loaded dendrimer-gold nanostructure. The dendrimer-gold hybrid was created by combining AuCl_4_^-^ ions with PEGylated amine-terminated generation 5 poly(amidoamine) dendrimers. To achieve targeted binding to colorectal adenocarcinoma cells, the system was conjugated with the MUC-1 aptamer. The results demonstrated the accumulation of the theranostic agent in HT29 and C26 cells, showing greater toxicity compared to the non-targeted system. Moreover, *in vivo* experiments showcased the high potential of the theranostic system in CT-scan tumor imaging and cancer therapy. Twelve hours after the administration of Apt-PEG-AuPAMAM-CUR, the CT scan images of the treated group exhibited a higher signal value in the tumor tissue. These findings highlight the efficacy of this therapeutic platform and its considerable potential in combating colorectal cancer adenocarcinoma 131.

### Carbon-based nanomaterials

Carbon nanomaterials, including carbon nanotubes, graphene and graphene oxide (GO) and their hybrids, have been extensively researched due to their exclusive properties, which make them useful for imaging and biomedical applications 132. Carbon nanomaterials can be paired with aptamers to act as electrochemical sensors for cancer diagnosis and treatment when appropriately functionalized. For instance, a recent study established a graphene-hemin nanosystem comprising gold NFs with high catalytic activity. When aptamers that connect to K562 leukemia cancer cells were introduced, the nano platform could detect the target cells with high sensitivity. Additionally, aptamer-based graphene nanomaterials can be used to analyze cell membrane surface and intracellular biomarkers 133. A multifunctional theranostic platform has been developed, utilizing the conjugation of porphyrin (P) derivatives with high oxygen production activity, aptamer-functionalized graphene quantum dots (GQDs), and PEG. This platform has exhibited favorable compatibility and low toxicity. Notably, the intrinsic fluorescence of GQDs enables the differentiation between cancer cells and somatic cells. Additionally, the high surface area of the platform facilitates gene delivery for the detection of cancer-related microRNA (miRNA). Furthermore, this system demonstrates remarkable photothermal conversion efficacy, reaching up to 28.5%, along with a high quantum yield of oxygen production. Consequently, it proves to be suitable for progressive photothermal and photodynamic therapies 134.

### Metal-organic framework-based nanomaterials

MOFs are a group of coordination nanomaterials with a range of unique properties and can be used in various fields, including catalysis, biosensors and biomedical applications 8, 135. MOFs can be easily functionalized and have a high capacity for the cargo loading, making them suitable for combining with aptamers. As a result, different aptamer-based MOF platforms have been developed for cargo delivery in cancerous tissues 136. For instance, aptamers that bind to specific molecules can be intended to act for controlling delivery. Researchers created ATP-stimulate nanoparticles comprising nanoscale MOFs (NMOFs) to target the delivery of fluorescent molecules 137. The nanoparticles were fabricated with complementary nucleic acids that hybridized with an aptamer to lock the nanoparticle and prevent cargo leakage. After accumulating at the tumor area, the nanoparticles were unlocked through ATP to deliver cargoes. The ATP-responsive NMOFs nanoparticles were also modified with AS1411 aptamers to give them targeting ability and were shown to precisely deliver the drug to prevent cancer cell growth. Another type of MOF-based aptamer can be focused on cell recognition and delivery. Ning and co-workers developed a surface coordination chemistry approach for efficiently immobilizing functional DNA on the surface of NMOFs, which allowed for the targeted delivery of therapeutic DNA 138. On the other hand, researchers created porphyrinic metal-organic framework (ZrMOFs) nanoparticles for imaging by functionalizing the nanoparticles with phosphate-terminal DNA aptamers. This enabled the ZrMOF nanoparticles to accumulate in cells selectively and allowed for targeted imaging and increased PDT. This strategy may provide new approaches for functionalizing other types of MOF nanomaterials 139.

In another study, a unique type of material called a bimetallic MOF was created by combining different MOFs to form a hybrid. This method, called MOF-on-MOF, allows for the properties of both MOFs to be incorporated, resulting in a material with new, distinct characteristics. Researchers utilized this new material, specifically the bimetallic CuZr-MOF, to support immobilising a biomolecule called aptamer on the surface of an electrode, forming an electrochemical aptasensor. This sensor can detect a biomarker called miR-21 associated with cancer. Results exhibited that the properties of the CuZr-MOF could be tailored by varying the order of addition of the organic linkers. The aptasensor displayed a greatly sensitive and accurate diagnosis of miR-21, with a LOD of 0.45 zM. Additionally, the sensor presented exceptional specificity, and reproducibility, making it a highly effective tool for early and sensitive diagnosis of miRNA-related diseases. To evaluate the precision and sensitivity of the aptasensor, the detection and quantification limits were determined by exposing the Apt/CS-CuZr-MOF/GCE to a range of concentrations of miR-21 in PBS (pH 7.4). As the concentration of miR-21 increased, the current generated by the sensor decreased as more miR-21 strands bound to the aptamer strands, hindering the electron transfer to the electrode surface. The detection and quantification limits were determined using the square-wave voltammetry (SWV) technique. The results exhibited that the aptasensor had a linear response to miR-21 concentrations from 1 zM to 1 pM, with a high correlation coefficient (R2) of 0.99. The lowest miR-21 concentrations that could be detected and quantified by the aptasensor were 0.45 and 1.5 zM, respectively, which demonstrates its high sensitivity and precision 140.

In a research effort, scientists have developed a method for targeted anti-tumor drug delivery using a unique combination of materials. They utilized zirconium-based MOFs (Zr-MOF) embedded with silver nanoclusters (Ag NCs) and employed an aptamer called AS1411 as a guide to target cancer cells. The resulting system was called UiO-66@AgNCs@Apt. They also created a variant of this system, called UiO-66@AgNCs@Apt@DOX, which incorporated the cancer drug DOX and was designed to show increased loading efficiency of around 90% and controlled release of the drug for up to 96 hours. The study exhibited that the aptamer-modified delivery system could effectively target and be taken up through cancer cells with over 80% specificity using confocal laser scanning microscopy. They also tested the system on cancerous and non-cancerous cells and found that the drug is effectively delivered to cancer cells, specifically with a cellular uptake of around 30% more. The system shows a robust enhancement of anti-tumor effect with low cytotoxicity in an extensive range of concentrations from 5-50 µg/mL, making it a promising candidate for controlled drug delivery in cancer therapy. Scientists studied the ability of UiO-66@AgNCs@Apt/DOX to selectively kill cancer cells and spare normal cells using an *in vitro* assay, comparing their effectiveness on MCF-7 cells to normal L929 cells. They found that both formulations had low toxicity at high concentrations. Still, the UiO-66@AgNCs@Apt/DOX composite was more effective at killing cancerous and normal cells with an inhibition rate of 73.3% and 64.4%, respectively, at 5 µg/mL. The one-pot encapsulated UiO-66@AgNCs@Apt@DOX had greater cytotoxicity on MCF-7 cells with an inhibition rate of 80.3% at 10µg/mL; however, it had a lower rate of 54.9% on L929 cells at the same concentration. This discrepancy may be due to the pH and environment within the endosomal compartment of cancer cells and the specificity of aptamer modification that enhance the targeted binding to MCF-7 cells by specific internalization. These results indicate that UiO-66@AgNCs@Apt@DOX has a higher potential for the selective cancer cell 141.

Researchers have successfully developed a novel method for imaging and drug delivery targeted specifically towards triple-negative breast cancer, a form of cancer commonly treated with chemotherapy. This breakthrough was achieved through the creation of a unique nanocarrier, referred to as Fe_3_O_4_@MOF-DOX-CDs-Apt, consisting of an anti-cancer drug, fluorescent carbon dots (CDs), and an aptamer. The nanoplatform is constructed by combining a nucleolin-DNA aptamer with a magnetite core and a MOF shell. For fluorescence imaging purposes, CDs are encapsulated within the Fe_3_O_4_@MOF nanocomposite, thereby imparting fluorescence properties. The resulting Fe_3_O_4_@MOF nanostructures exhibit a monodisperse morphology and possess a size of 17 nm. The nanocarrier would release its drug payload specifically in the existence of certain cancer cells, which overexpress a protein called nucleolin. The release process was pH dependent, allowing for more efficient drug delivery. These nanocarriers are more effective in targeting cancer cells with a specificity rate of over 85% compared to normal cells. They also exhibited fluorescence imaging capabilities, which can be used to monitor their distribution in the body. Cytotoxicity experiments showed that the carriers inhibited cancer cell proliferation and induced apoptosis, with over 77% of MDA-MB-231 cancer cells killed after 24 hours of incubation. The same concentration of the nanocarrier has less than 10% impact on normal HUVEC cells. Therefore, the researchers propose that these nanocarriers could be a potential solution for treating triple-negative breast cancer through their ability to deliver drugs and image their distribution. The studies suggest that several mechanisms work together to create a multi-stimuli-responsive drug delivery system. One such mechanism is using acid-sensitive UiO-66 MOFs, which can release drugs in low-pH environments found in cancer cells. This process was enhanced by modifying the MOF with amino groups, making it more sensitive to protonation at a pH below 6.3. Additionally, aptamers were used to lock the pores of the MOF, only releasing drugs when they bind to a specific target, such as a protein overexpressed on MDA-MB-231 cancer cells. The system also includes a magnetic core, which could have the potential for magnetic-responsive drug delivery, although this aspect still needs to be fully studied in these experiments 142.

### Magnetic-based nanomaterials

**Figure 5:**
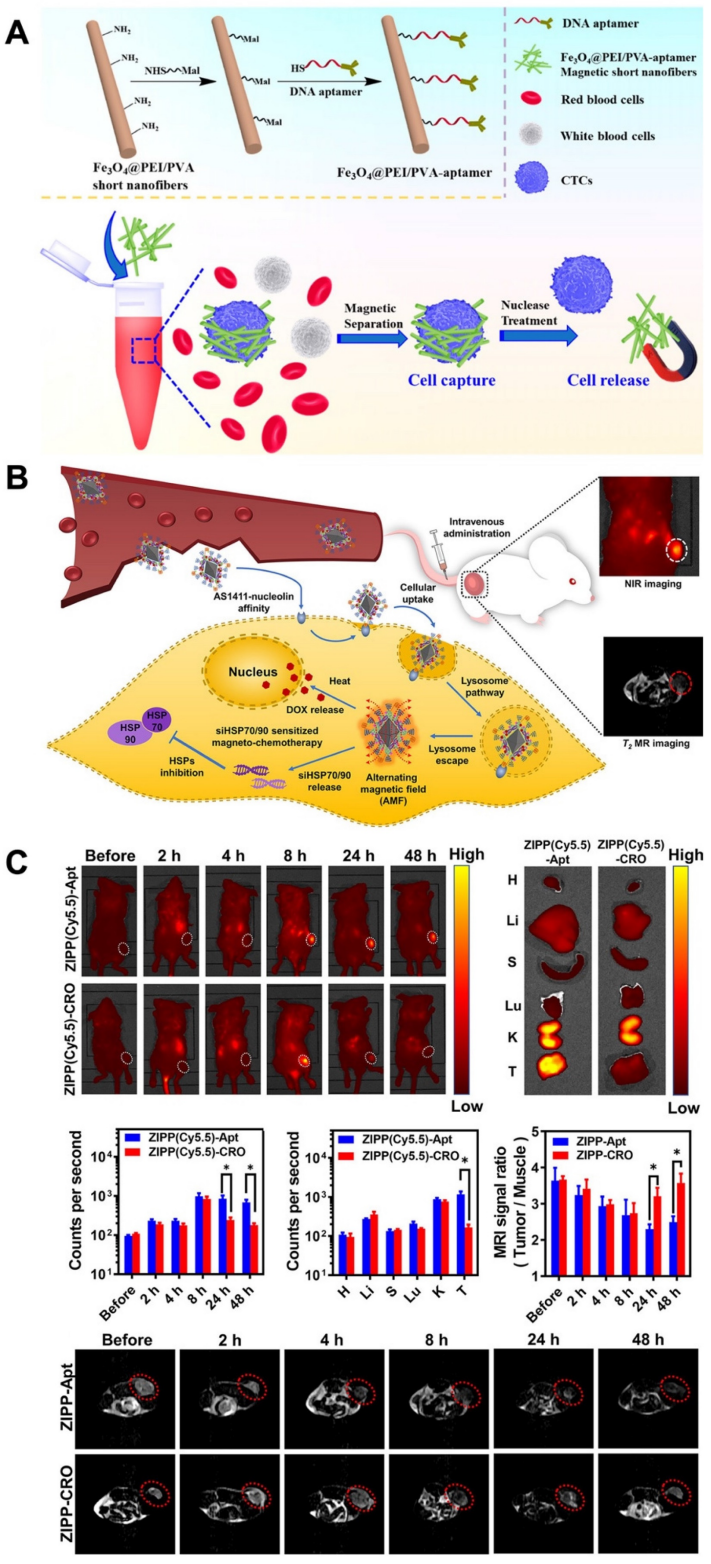
Design of DNA Aptamer- Magnetic Nanofibers for Effective Capture of CTC. (A) schematic shows the surface modification of MSNFs for the capture of cancer cell. Reprinted (adapted) with permission from 144. Copyright 2019 American Chemical Society. Sensitized magneto-chemo theranostics and NIR/MR dual-modality imaging. (B). Accumulation of the ZIPP with AS1411 in the tumor after 8 h. (C) The MRI signal ratio of tumor to the muscle is reliable with the accumulation rate detected from NIR imaging that ZIPP-Apt was efficiently reserved in the tumor area and triggered a signal decrease at 24 and 48 h after injection. Reprinted (adapted) with permission from 149. Copyright 2021 American Chemical Society.

Magnetic nanomaterials with functional magnetic abilities can be used in biosensors, delivery systems, biosensing systems and the separation of specific cells. Promising physiochemical properties and the ability to accommodate targeting moieties make superparamegnetic iron oxide nanoparticles (SPIONs) appropriated as theranostic agents 143. Aptamer-functionalized magnetic nanomaterials, which have high selective recognition and binding abilities, have become influential in capturing and separating biological samples. One recent example of aptamer-functionalized magnetic nanomaterials being used for cell isolation is the detection of CTCs for clinical diagnostic purposes. Polyethylenimine (PEI)- stabilized Fe_3_O_4_ nanoparticles encapsulated inside PEI/poly(vinyl alcohol) nanofibers. After a treatment needed for the magnetic short nanofibers (MSNFs), surface conjugation of the aptamer was done. The aptamer-MSNFs, with a size of 350 nm, showed the capturing cancer cells with an efficacy of 87% and allowed the release of cancer cells with a high efficacy of 90% after nuclease treatment. Especially, this aptamer-MSNFs showed a critically greater release efficacy compared to the commercial magnetic beads 144 (Figure 5A).

Ding et al. developed a nanoplatform that used near-infrared (NIR) Ag_2_S dots with aptamer modification and the encapsulation of magnetic nanoparticles in a cell membrane to efficiently isolate and detect CTCs. The nano-bio-probe had a great capture efficiency of 97 % and purity for CTCs of 96% and could also be applied to detect CTCs in blood samples. Researchers established a technique for capturing and releasing CTCs using aptamer-based magnetic nanofibers. Aptamer-based magnetic nanomaterials have also been discovered for the separation of CD8^+^T cells. Researchers produced a DNA aptamer based on SELEX and used it to separate CD8^+^T cells at high yields with properties. This demonstrates the effective potential of aptamer-based magnetic nanoparticles in the traceless isolation of lymphocyte subsets 144-148.

In another study, researchers developed superparamagnetic iron oxide nanoparticles (SPIONs) coated with gold nanoparticles (Au NPs) for the purpose of magnetic resonance imaging (MRI) and photothermal therapy of colon cancer cells. The formation of SPIONs was achieved through a microemulsion method. The inclusion of Au NPs served to reduce the cytotoxicity of SPIONs and enhance their photothermal capabilities. To act as a targeting agent, the thiol-modified MUC-1 aptamer was conjugated onto Au@SPIONs, allowing for binding and synergistic affinity. MTT results demonstrated that the nanostructure exhibited minimal toxicity within the concentration range of 10-100 μg/ml, indicating lower cytotoxicity compared to bare nanoparticles. MR imaging revealed significant contrast enhancement in vitro, indicating that SPIONs could be utilized as effective contrast agents. Furthermore, cells treated with Apt-Au@SPIONs exhibited a higher death rate compared to the control group when subjected to near-infrared (NIR) irradiation. These developed nanomaterials hold promise as theranostic agents for MR imaging and photothermal therapy of colon cancer cells 143.

However, the combination of chemotherapy with magnetic hyperthermia holds promise as a strategy for cancer therapy. Nonetheless, the nonspecific accumulation of magnetic nanoparticles has limited their applications. To address this, researchers have developed a highly selective theranostic nanosystem called ZIONO-PAMAM-PEG (ZIPP)-Apt:DOX/siHSPs, designed for theranostic drug/gene delivery and magnetic resonance imaging-guided magnetochemotherapy. The cellular uptake of the nanoplatforms has been significantly enhanced through the AS1411-nucleolin affinity, while also achieving a simultaneous reduction of HSP70/90 to sensitize magnetic hyperthermia and chemotherapy. Following intravenous injection, the nanoplatform successfully accumulates in tumor areas as confirmed by NIR and T2-weighted MR dual-modality imaging (Figure 5B-C). To ensure lysosome escape, dendrimers with proton sponge properties were utilized. Furthermore, the downregulation of HSP70/90 via siRNAs sensitized cancer cells to hyperthermia and chemotherapy. Intriguingly, intracellular hyperthermia was stimulated, leading to the rapid delivery of therapeutic drugs such as DOX and resulting in HSP70/90 exhaustion that further sensitized magnetochemotherapy. This study demonstrates the promising potential of magnetic nanoparticles in theranostic drug and gene delivery, as well as their application in imaging and magnetochemotherapy for cancer treatment 149. Table 1 showed several recent studies about the role of aptamer-based nanomaterials in theranostic applications.

## Conclusion and future perspectives

Aptamer-based nanomaterials have revealed exclusive capabilities in diagnostic and therapeutic applications and have been extensively studied. This review discusses some recent developments in biological applications of DNA nanomaterials embedded in aptamers. Aptamers' ability to recognize and bind to specific targets, from molecules to cell lines, has contributed significantly to the development of aptamer-based nanomaterials in biomedical fields, such as targeted cell imaging and drug delivery, and cancer diagnosis and therapy. Aptamers and DNA nanostructures, both advanced platforms for biomedical applications, have unique properties that are utilized in various fields. However, they demonstrate even more potential when integrated into areas such as biosensing, imaging and targeted drug/gene delivery.

Biosensors that utilize aptamer-integrated DNA nanostructures possess a unique combination of ultra-high sensitivity and specificity. The precise addressability of DNA nanostructures allows for control of the sensor's physicochemical properties, while aptamers, known for their binding specificity and affinity, allow for accurate sensing of a wide range of targets, from small molecules to entire cells. Signal transduction methods in these sensors are diverse, including options such as electrochemistry, fluorescence, atomic force microscopy and visual readout for point-of-care tests. In the field of bioimaging, aptamer-integrated DNA nanostructures are utilized extensively. The compatibility of DNA nanostructures, and enzymatic resistance make it an ideal option. Additionally, aptamers selected by cell-SELEX can discriminate positive target cells from normal controls, allowing for the quantitatively or dynamic monitoring of biospecies, including membrane biomolecules, ATP, metal ions, and environmental factors that may reside on cell surfaces, inside cells or in living bodies. The exceptional specificity against cancer cells, stability in biofluids and tissue penetration of aptamer-based DNA nanostructures make them perfect carriers for targeted drug delivery. Diverse therapeutics agents can be anchored or encapsulated specifically with high payload in DNA nanostructures for enhanced or synergistic therapy. Additionally, dynamic DNA nanotechnology has enabled the development of smart nanodevices, which are now being explored for applications in drug delivery and biological process.

Despite its remarkable progress, aptamer-integrated DNA nanostructures still face limitations that need to be addressed. One of the main challenges is screening high-performance aptamers, which is a complex, time-consuming, and labor-intensive process. On the other hand, the SELEX technique is time-consuming and labor-intensive. Therefore, researchers are searching for new, simpler methods of aptamer selection. The competitive non-SELEX selection utilizes the idea that aptamers can be selected for a target if two similar targets exist simultaneously. In this technique, PCR is not needed, and specific aptamers can be selected. Researchers were able to successfully select aptamers for influenza subtypes using this method 157.

Another challenge lies in the stability of both aptamers and DNA nanostructures, which need improvement for intracellular and *in vivo* applications. For targeted drug delivery, aptamers need protection from the physiological environment, while the integrity of DNA nanostructures must be maintained during transit. Efforts have been made to enhance stability through engineering aptamers and DNA nanostructures, as well as employing modified nucleic acids. However, further research is required to validate their efficacy. Moreover, *in vivo* applications raise concerns regarding unwanted "gene regulation" since DNA strands within DNA nanostructures can potentially interact with mRNAs or genes. Therefore, conducting toxicity assessments of aptamer-based DNA nanostructures is crucial. Furthermore, most studies on aptamer-based nanomaterials have only been tested at the animal level, necessitating further research to ensure their safety for use in clinical trials. This involves studying their toxicity, impact on genomics, and in vivo safety. Additionally, investigating the physicochemical interface between aptamers and nanomaterials during the construction process is essential to enhance compatibility and reduce off-target effects. In the future, improvements to aptamer-based nanomaterials may involve increasing the fluorescence excitation/emission wavelengths of nanomaterials to enhance in vivo imaging and therapy. Clever surface modifications and reconstructions of aptamers can also be explored to enhance the binding capability and stability of aptamer-embedded nanomaterials for in vivo applications. As chemistry and materials advance, aptamer-embedded nanomaterials are likely to find increased usage in diagnostic and therapeutic applications. Additionally, the practical use of DNA nanostructures is hindered by their high cost and low purity. Another challenge pertains to the utilization of aptamer biosensors in complex samples for point-of-care applications. Hence, the transfer of aptamer-based sensors from the laboratory to biomedical applications still encounters certain challenges.

While aptamer-integrated DNA nanotechnology has made significant progress, there are still challenges to be overcome. Researchers are working to simplify aptamer selection, enhance the stability of aptamers and DNA nanostructures, and address concerns about gene regulation. On the other hand, aptamers and nature-inspired methods together on a microfluidic chip can enhance the diagnosis and long-term monitoring of patients. Regarding therapy, aptamers and nature-inspired methods, including exosomes, have a key-lock relationship, where aptamers target specific cells for drug delivery. However, the selection process for aptamers is complicated, and the mechanism of those nature-inspired methods, including exosomes and lipid-based vesicle uptake by target cells, is not fully understood. Research on nucleic acid structures and scalability is needed to improve aptamer-nature-inspired therapy. Scalability is particularly important to make this technology more widely available in clinical settings. Research on optimizing conditions for aptamer-nature-inspired targeting is also required to achieve maximum efficiency and specificity.

Despite these challenges, the field is advancing and increasing attention and resources are being devoted to this field, which will accelerate its development and expand the role of DNA nanostructures in biology and medicine. The integration of aptamers and DNA nanostructures has the potential to revolutionize the field of biology and medicine. Their unique properties and versatility allow for a wide range of applications, and new and exciting applications will continue to be discovered in the future.

## Author contributions

N.R., S.C., S.A., and R.N.V. wrote the main manuscript, conceptualized and finalized the manuscript. All authors reviewed and approved the manuscript.

## Figures and Tables

**Table 1:** Different types of aptamer-based nanomaterials in multiple theranostic applications

Nanomaterials	Aptamers	Conjugation method	Theranostic applications	Descriptions	Ref
ALGDG2	AS1411 aptamer	Covalent amide bonds	Cancer therapy	AS1411-liposome-PEG-MnO-PTX showed the potential of simultaneous MRI diagnosis and therapy of renal carcinoma, strong MR contrast effect in the tumor, high half-life during circulation in blood, and high potential in tumor growth inhibition.	150
SPIONs)/ poly(lactic-*co*-glycolic acid) (PLGA)	AS1411 aptamer	Covalent carboxy linkage	Cancer imaging and therapy	Aptamer conjugated nanoparticles increased cellular uptake of DOX in C26 cancer cells, increased. the cytotoxicity effect of drug and greater tumor inhibition in mice bearing C26 colon carcinoma xenografts.	151
PAMAM dendrimer	AS1411 aptamer	Amide condensation reaction	Bioimaging and drug delivery	This platform act as a dual function of targeting and drug delivery for in vitro and in vivo imaging and cancer therapy, with high affinity of aptamer AS1411 toward cancer cells, and controlled delivery of DOX into cells multifunctional nano-drug delivery systems for precise cancer theranostics.	152
Liposomes	AS1411 aptamer	Covalent coupling reaction	MRI diagnosis and cancer therapy	AS1411 aptamer can increased the MRI effect and the tumor growth inhibition, presenting its potential as a theranostic agent for renal carcinoma.	153
QDs-	Anti-EGFR aptamer	N/A	Theragnosis of triple-negative breast cancer (TNBC) targeted drug delivery	EGFR- QLs exhibited increased delivery to target cancer cells, more effective gene silencing and increased tumor imaging.	154
Au@SPIONs	MUC-1 aptamers	Thiol-maleimide reaction	MR imaging and photothermal therapy of cancer cells	Aptamer-Au@SPIONs were revealed to have greater uptake in MUC-1 positive cells and higher toxicity than other materials.	143
Au@Ag/Au nanoparticles	S6 aptamer	Thiol-maleimide reaction	Imaging and specific PTT cancer therapy	S6-Au@Ag/Au nanoparticles could effectively internalization into the A549 cells, and destruct cells under the NIR irradiation.	155
PEG-AuPAMAM-CUR	MUC-1 aptamer	Covalent conjugation	CT-scan tumor imaging and cancer therapy	This system showed greater cellular uptake, internalization and high cytotoxicity in C26 and HT29 cells.	131
PEGylated-MoS_2_/Cu_1.8_S	AS1411 aptamer	N/A	Chemo-Photothermal cancer Therapy/ photoluminescence imaging (PLI)	This theranostic nanosystem were revealed to have targeted delivery, excellent photothermal conversion efficiency, and antitumor efficiency.	156

## Abbreviations

Ag NCs: silver nanoclusters
AMD: age-related macular degeneration
Apt: Aptamer
aptNTrs: aptamer-tethered DNA nanotrains
ASO: antisense oligonucleotide
atcHCR: aptamer-triggered clamped hybridization chain reaction
BMSCs: bone marrow mesenchymal stem cells
cDNA: complementary DNA
CDT: chemodynamic therapy
cSELEX: circular SELEX
CTCs: circulating tumor cells
ds: double-stranded
DOX: doxorubicin
D-PGM: DNA nanoscale precision-guided missile
EPR: enhanced permeability and retention
FE-SEM: field emission-SEM
GC: guidance/control
GQDs: graphene quantum dots
LAL: Limulus amebocyte lysate
MRI: magnetic resonance imaging
Mod-SELEX: modified SELEX
MOFs: metal-organic frameworks
MSNFs: magnetic short nanofibers
NA: nucleic acids
NCP: nanostructured coordination polymer
NFs: nanoflowers
NIR: near-infrared
NMOFs: nanoscale metal-organic frameworks
NW: nanowire
ONV: oligonucleotide nanovehicle
PCR: polymerase chain reaction
PDT: photodynamic therapy
PEG: polyethylene glycol
PEI: polyethylenimine
PSMA: prostate-specific membrane antigen
RCA: rolling circle amplification
RET: reorganized throughout transfection
ROI: regions of interest
SELEX: systematic evolution of ligands by exponential enrichment
siRNA: short-interfering RNAs
ss: single stranded
STEM: scanning transmission electron microscope
SPECT: single-photon emission computed tomography
SWV: square-wave voltammetry
TEM: transmission electron microscopy
TPGS: D-ɑ-tocopheryl polyethylene glycol succinate
UCNP: upconversion nanoparticle
VEGF: vascular endothelial growth factor
WH: warhead
YMA: Y-shaped monomer A
YMB: Y-shaped monomer B
Zr-MOF: zirconium-based MOFs
